# Assessment of the Influence of Various Concentrations of Sodium Hypochlorite on Stem Cell Derived From Human Exfoliated Deciduous Teeth (SHED) Proliferation and Differentiation

**DOI:** 10.7759/cureus.33024

**Published:** 2022-12-27

**Authors:** Viral Maru, Ashwini KB, Manisha Madkaikar, RK Sarada Devi, Ashita Gada, Salil Bapat

**Affiliations:** 1 Pediatric Dentistry, Government Dental College and Hospital, Mumbai, IND; 2 Dentistry, Bangalore Institute of Dental Sciences & Research Centre, Bangalore, IND; 3 Immunology, National Institute of Immunohaematology, Mumbai, IND; 4 Dentistry, Regional Institute of Medical Sciences, Imphal, IND; 5 Public Health Dentistry, Sau. Mathurabai Bhausaheb Thorat (SMBT) Dental College and Hospital, Ghulewadi, IND

**Keywords:** naocl, odontogenic differentiation, proliferation, sheds, endodontic regeneration

## Abstract

Objectives

Previous research has established that sodium hypochlorite (NaOCl) has a detrimental effect on dental stem cell viability and maturation. However, a review of the literature revealed no study evaluating the response of NaOCl to the survival of stem cells derived from human exfoliated deciduous teeth (SHEDs). Hence, the aim of the present trial was to assess the influence of various dilutions of NaOCl on SHED proliferation and differentiation.

Materials and Methods

The 5.25% NaOCl solutions at concentrations of 0.5, 0.1, 0.025, 0.0125, and 0.005 mg/ml were used to assess the response to SHED proliferation and differentiation through methyl tetrazolium (MTT) assay, alkaline phosphatase (ALP) assay, and reverse transcription polymerase chain reaction gene expression analysis at various time point intervals.

Results

MTT assay showed that the viability of SHEDs decreased with an increase in the concentration of NaOCl and an increase in incubation time. The ALP activity decreased with an increase in the concentration of NaOCl up to 14 days of incubation. However, the ALP activity of all the test specimens further decreased after 14 days of incubation. The gene expression levels of osteocalcin, dentin sialophosphoprotein, and STRO-1 were statistically significant when compared to the control after one, three, and seven days of incubation.

Conclusion

Different doses of NaOCl other than 0.5 mg/ml revealed encouraging outcomes in terms of proliferation, long-term ALP functioning, and odontogenic differentiation potential when cultivated in SHEDs.

## Introduction

Endodontic instruments and irrigants are used to perform biomechanical preparation during pulpectomy. These irrigant solutions are used to dissolve organic and dentinal debris and sterilize and lubricate the canal system, during biomechanical preparation [1]. Sodium hypochlorite (NaOCl) is frequently used to irrigate root canals at levels ranging from 0.5 to 6.15%. While its solvent and antiseptic qualities are enhanced at larger concentrations, so are its hazardous consequences [2]. Although NaOCl has been the most frequently used chemical for endodontic irrigation, there is no universal agreement on the dosage that produces the best outcomes without causing adverse symptoms [3]. Sirtes et al. reported that 1% NaOCl is adequate to disintegrate pulp tissue [4], whereas the literature reports that NaOCl is typically employed at levels ranging from 0.5 to 5.25% and is most frequently used in regenerative endodontic procedures due to its antibacterial properties and superior tissue dissolving capabilities [5].

Previous research has established that NaOCl has a detrimental effect on dental stem cell viability and maturation [6]. Additionally, NaOCl dramatically reduced the proliferation of dental pulp stem cells (DPSCs) in a dilution-dependent mode [7]. In vivo investigations demonstrated that exposure to 5.25% NaOCl severely impairs DPSC maturation into odontoblast cells. Additionally, 3% NaOCl inhibited dentin sialophosphoprotein (DSPP) gene expression by 50% [8]. According to Liu et al., NaOCl decreased the survival and adenosine triphosphate (ATP) levels of DPSCs, stem cells derived from the gingiva, and periodontal ligament in a concentration- and time-dependent way [9]. Three percent NaOCl is not only the most preferred endodontic irrigant during pulpectomy but also gaining popularity as a pulpotomy medicament in primary teeth [10,11,12]. Hence, NaOCl should possess adequate biocompatibility to promote pulp healing and stem cell activity.

Stem cells derived from human exfoliating deciduous teeth (SHEDs) have the capacity to induce bone formation, generate dentin, and differentiate into other non-dental mesenchymal cell derivatives in vitro. In contrast to DPSCs, SHEDs exhibit a higher proliferation rate, increased population doublings, osteoinductive capacity in vivo, and an ability to form sphere-like clusters [13]. However, a review of the literature revealed no study evaluating the response of NaOCl to the survival of SHEDs. The goal of this study was to see how varying NaOCl concentrations affected SHED cell proliferation and differentiation into odontogenic cells.

## Materials and methods

The current in vitro experimental trial adhered to the 2021 standards for reporting research given by Nagendrababu et al. [14]. The sample size for this experiment was estimated using the "resource equation" strategy [15]. All analyses were carried out in triplicate for each test specimen and consequence.

Preparation of test specimens

The cell proliferation and differentiation of SHEDs were investigated with 5.25% NaOCl at doses of 0 (control), 0.5, 0.1, 0.025, 0.0125, and 0.005 mg/ml.

Isolation and characterization of SHEDs 

The Institution Research & Ethical Board (IREB/2021/GDC/01) approved the current trial. The parents or guardians of children aged six to nine years who visited the Pediatric & Preventive Dentistry department (Government Dental College & Hospital, Mumbai) completed an informed consent form permitting the exfoliating primary teeth (n = 8) to be included in the research. The extracted teeth were properly rinsed with normal saline and transported to India's National Institute of Immunohaematology in Parel, Mumbai for SHED isolation. To isolate the pulp tissues from the teeth, barbed broaches were utilized. Hank's Balanced Salt Solution (Gibco, USA) was used to rinse the pulp tissues. Following that, collagenase-A (3 mg mL^-1^) was utilized for 60 minutes of enzymatic digestion at 37 °C. Cells (1.5 x 104 cells/cm^2^) were then placed in 25-cm^2^ plastic culture flasks and cultured for three days in a complete medium (Dulbecco's modified Eagle’s medium (DMEM) at 37 °C & 5% CO_2_).

Red blood cells and other non-adherent cells were discarded. To aid with growth, new media were used. P0 was defined as the expansion of adhering cells to 80% confluence. To clean the cells, phosphate buffer saline (PBS) was employed, and cells were detached through culturing for 2-5 minutes at 37 °C with 0.25% trypsin. DMEM was added to inhibit trypsin activity. SHEDs were centrifuged at 500 g for five minutes and seeded with 5 x 103 cells cm^2^ in 75-cm^2^ flasks. Prior to the trials, the phenotypes of SHEDs were determined using a flow cytometer with specific antibodies against a cluster of differentiation (CD) 45, CD 73, CD 105, CD 34, CD 90, and human leukocyte antigen (HLA-DR) (BD Biosciences, Pharmingen, USA) [16].

Cell viability assay

A 96-well plate was seeded with 1x104 cells/well and then cultured for one day to help the cells adhere to the bottom of the wells. After aspirating the culture medium from each well, 150μl of sterile NaOCl solution at varying concentrations was added. Each test specimen was incubated for two hours, four hours, eight hours, and 24 hours. Then, 10μl of 3-(4,5-dimethylthiazol-2-yl)-2,5-diphenyl tetrazolium bromide (MTT) reagent was applied to each well (Cayman Chemical Company, Ann Arbor, USA). Following that, 96-well plates were cultured for an additional three hours at 37°C. Finally, after aspirating the solution from each well, 150μl of the dissolving agent was added to dissolve the formazan precipitate. Using a microplate reader (BioTek®, Winooski, USA), the absorbance of each sample was measured at 570 nm, using absorbance at 690nm as a reference wavelength. The specimens that revealed a cell viability of less than 30% were regarded as cytotoxic [17].

Alkaline phosphatase (ALP) assay 

SHEDs were counted and grown overnight in 24-well plates in complete media (2 x 104 cells/well). SHEDs were cultivated in complete media (DMEM) with test specimens the next day, while the control group was cultured in complete medium (DMEM) only. ALP activity was examined after seven, 14, and 21 days of incubation. SigmaFast p-nitrophenyl phosphate tablets (Sigma-Aldrich, Burlington, USA) were used to measure ALP activity. SHEDs were cultured in active dissolved solution for 60 minutes at 370 C (in the dark) after two PBS washes. Two hundred microliter solution from every well was introduced into a 96-well plate, and AB405 nm was assessed with an enzyme-linked immunosorbent assay plate reader (BioTek®, Winooski, USA). The absorbance readings were transformed into ALP moles using a calibration curve built with known ALP moles [18].

Gene expression analysis

SHEDs were seeded in a six-well plate (1x105 cells/well). The cells were treated with different concentrations of NaOCl at various time intervals of incubation. After three PBS washes, the medium was changed with differentiation-induction media consisting of minimum essential medium (MEM) plus 10% fetal bovine serum supplemented with dexamethasone (10 nM) (Sigma-Aldrich, Burlington, USA). The groups that were not treated served as controls. After one, three, and seven days of incubation, cells were harvested in Trizol. cDNA synthesis was conducted according to the manufacturer guidelines using the PrimeScriptRT reagent kit (Takara, Dalian, China). As an internal control, glyceraldehyde 3-phosphate dehydrogenase (GAPDH) was utilized. The gene expression was analyzed by polymerase chain reaction (qPCR) (StepOne Applied Biosystems, ThermoFisher Scientific, Waltham, USA) using TaqMan chemistry and predesigned primers and probe sets (Gene Expression Assays, Applied Biosystems, ThermoFisher Scientific, Waltham, USA). The sequences of primers are listed in Table 1. The levels of mRNA expression were determined with the ΔΔCt method (fold expression = 2 - (ΔΔCt ± SD)) [19].

**Table 1:** The primer sequences used in real-time RT-PCR analysis RT-PCR: Reverse transcription polymerase chain reaction

Gene name	Forward sequence	Reverse sequence
Dentin sialophosphoprotein	TTAAATGCCAGTGGAACCAT	ATTCCCTTCTCCCTTGTGAC
Osteocalcin	TCTGACAAAGCCTTCATGTCC	AAATAGTGATACCGTAGATGCG
STRO-1	GAAGCTAAAGTGGATTCAGGAGTA	TAAGCAGGGGACCATTACA

Statistical analysis 

In order to summarize the data, the mean and standard deviation were calculated. The data were analyzed using IBM SPSS Statistics for Windows, Version 21 (Released 2012; IBM Corp., Armonk, New York, United States). Several groups were compared using one-way analysis of variance, followed by post hoc investigation to understand the least significant difference. At p<0.05, variations were declared statistically significant.

## Results

Characterization of SHEDs

Flow cytometric examination of SHEDs revealed the strong manifestation of the positive marker CD 73 (96.60 %), CD 90 (93.30 %), and CD 105 (31.90%). The progeny of SHEDs did not manifest negative markers CD 34 (3.30%), CD 45 (1.35%), or HLA-DR (1.55 %).

Cell viability assay 

**Figure 1:**
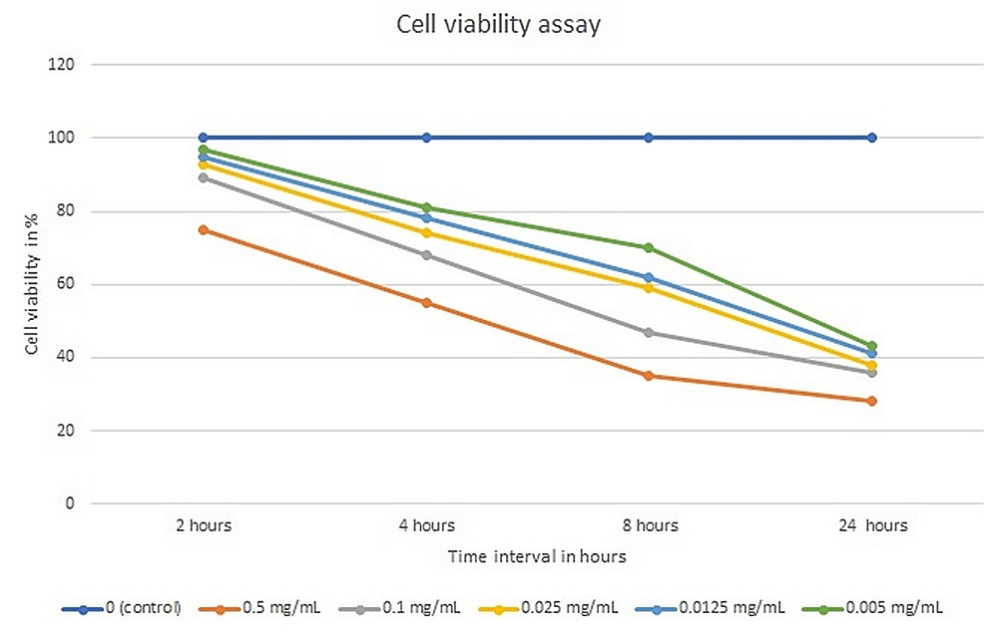
The cell viability percentage of all test specimens at different time intervals.

At all time points, the cell viability percentage of all samples was statistically significant (p = 0.01) compared to the control. On comparing with the control, a statistically significant pairwise comparison of p-values was reported for all samples, at all time intervals (p = 0.01). The highest cell viability percentage was observed with 0.005mg/ml (97 ± 1.4%) NaOCl followed by 0.0125 mg/ml (95 ± 1.8%), 0.025mg/ml (93 ± 5.0%),0.1mg/ml (89 ± 2.5%), and 0.5 mg/ml (75 ± 12.9%) after two hours of incubation. The cell viability for all test specimens reduced significantly as the incubation time interval increased from two hours to four, eight, and twenty-four hours (Figure 1). This trial reported that NaOCl at a concentration of 0.5 mg/ml (28 ± 6.1%) was cytotoxic after 24 hours of incubation.

ALP assay 

**Figure 2:**
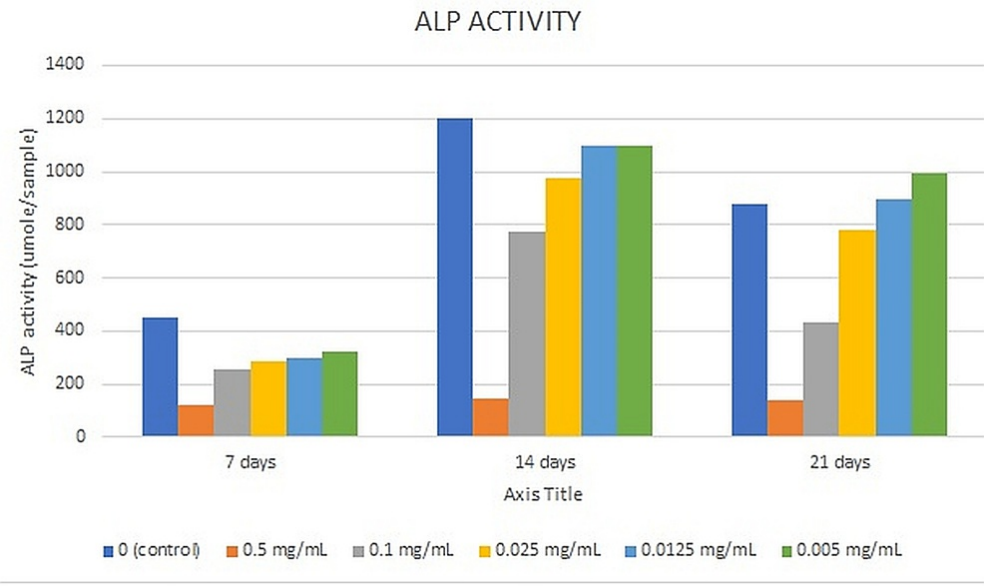
The ALP activity of all test specimens at different time intervals ALP: Alkaline phosphatase

At all time points, the ALP activity of all samples was statistically significant (p = 0.01) when compared to the control. A statistically significant pairwise comparison of p-values was reported for all samples compared to the control after seven, 14, and 21 days of incubation (p = 0.01). The highest ALP activity was observed with the control followed by following dilutions of NaOCl - 0.005 mg/ml (1100±258.19), 0.125 mg/ml (1095±328.36), 0.025mg/ml (975±344.82), 0.1mg/ml (775±11.05), and 0.5mg/ml (145±12.90) after 14 days of incubation. The ALP activity decreased significantly for all test specimens when compared to the control specimen after 21 days of incubation. (p < 0.05) (Figure 2).

Gene expression analysis 

**Figure 3:**
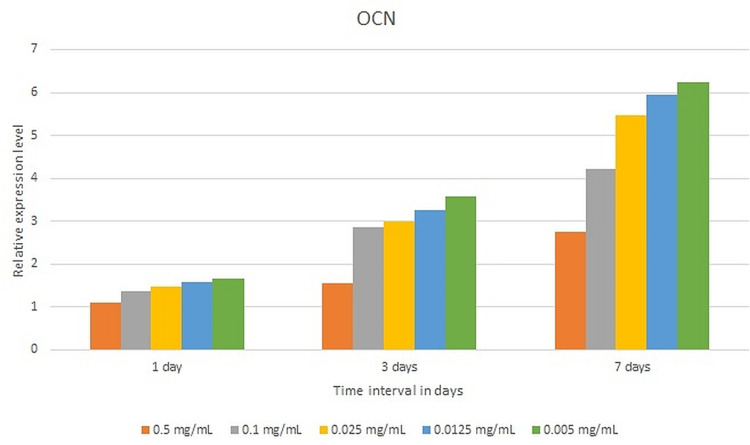
The expression levels of OCN in various test specimens after one, three, and seven days of incubation. OCN: Osteocalcin

The expression levels of osteocalcin (OCN) for SHEDs cultured in all samples as compared to the control were statistically significant at all time points. A statistically significant pairwise comparison of p-values was reported for all samples compared to the control after one-, three- and seven-day incubation (p = 0.01). The highest expression level was observed with 0.005 mg/ml (7.23±1.78), 0.125 mg/ml (±0.89), 0.025mg/ml (5.4±0.88), 0.1mg/ml (4.21±1.7), and 0.5mg/ml (2.76±1.78) after seven days of culturing (Figure 3).

**Figure 4:**
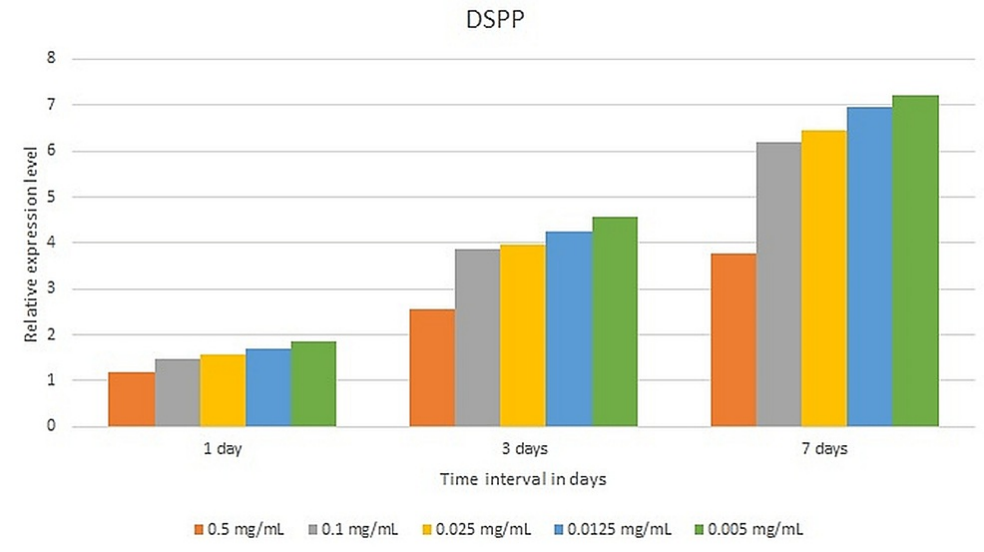
The expression levels of DSPP of various test specimens after one, three, and seven days of incubation. DSPP: Dentin sialophosphoprotein

The expression levels of DSPP for SHEDs cultured in all samples compared to the control were statistically significant at all time points. A statistically significant pairwise comparison of p-values was reported for all samples compared to the control after one, three, and seven days of incubation (p = 0.01). The highest expression level was observed with 0.005 mg/ml (6.23±0.89), 0.125 mg/ml (6.96±1.77), 0.025mg/ml (6.4±0.89), 0.1mg/ml (6.21±0.89), and 0.5mg/ml (3.76±0.89) after seven days of incubation (Figure 4). 

**Figure 5:**
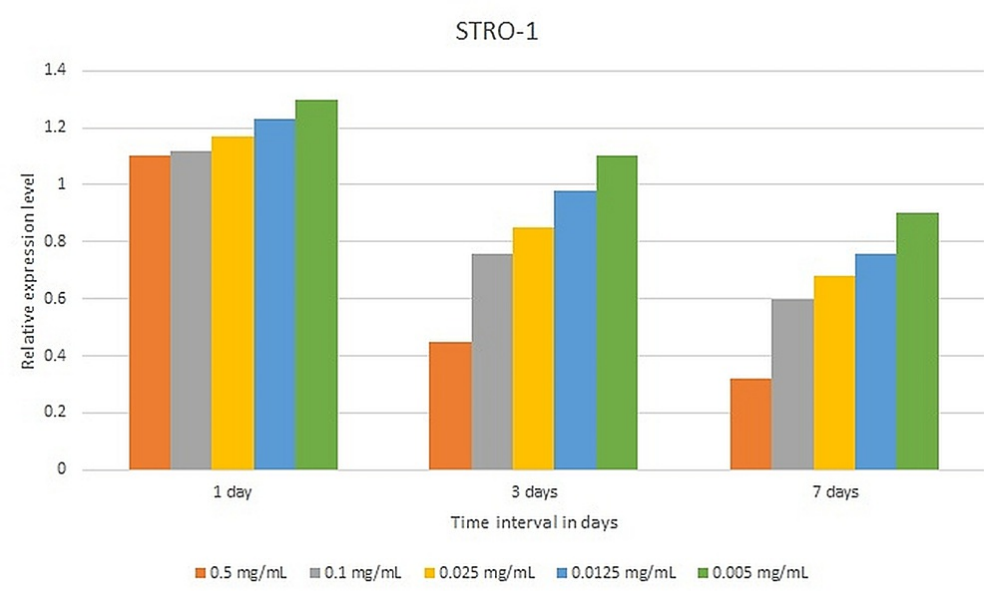
The expression levels of STRO-1 of various test specimens after one, three, and seven days of incubation.

The expression levels of STRO-1 for SHEDs cultured in all samples compared to the control were statistically significant at all time points. A statistically significant pairwise comparison of p-values was reported for all samples compared to the control after one, three, and seven days of incubation (p = 0.01). The highest expression level was observed with 0.005 mg/ml (1.3±0.17), 0.125 mg/ml (1.23±1.89), 0.025mg/ml (1.12±0.036), 0.1mg/ml (1.11±0.01), and 0.5mg/ml (1.06±0.13) after one day of incubation (Figure 5).

## Discussion

To irrigate the root canals during a pulpectomy, a variety of irrigating solutions with distinct constituents are used. These liquids may interact with the surrounding tissues, including the dentin and periapical tissues. A lack of biocompatibility in irrigation solutions can therefore result in the loss of cell growth, attachment, and enzyme systems in the region of contact. In this context, the assessment of the biocompatibility of pulpectomy materials is of great importance. Through the apical foramen, irrigation solutions do not restrict themselves to the root canal but also reach the periapical tissues, which consist of the cementum, periodontal ligament, and alveolar bone. Consequently, biochemical studies investigating the genetic impacts and toxicity of irrigation solutions on the tissues with which they come into contact are essential for limiting future risks to both the patient and the physician [20].

The hazardous impact is one of the most significant drawbacks of NaOCl, yet this irrigation solution is commonly employed by doctors and is the subject of several studies. NaOCl has been shown in several studies to be more cytotoxic and to cause more cellular injury than the control group and other test specimens. This toxicity increases linearly with longer exposure periods [6,7,21]. To corroborate these findings, we undertook this study to determine the osteogenic differentiation of SHEDs cultivated in varied concentrations of NaOCl over a range of time intervals. MSCs are supposed to express CD105, CD73, and CD90 in accordance with the International Society for Cellular Therapy's phenotypic standards. The phenotypic analysis of SHEDs in relation to the positive and negative markers utilized in this investigation was consistent with previous research [22,23,24]. Tetrazolium chemicals are used to provide a quantitative colorimetric test for determining the proliferation and survival of human cells. As a result, the MTT test has been widely employed to the point where it has become the de facto standard procedure for determining cell viability [17]. We discovered that the growth of NaOCl was related to the concentration employed in this investigation. This finding is consistent with previous research on the growth of stem cells cultivated in NaOCl. Essner et al. in 2011 reported that as the dilution of NaOCl raised from 0.041% to 0.3%, the cell proliferation reduced correspondingly when incubated in stem cells isolated from human permanent third molars [25]. Trevino et al. in 2011 also reported a similar finding when 6% NaOCl was exposed to stem cells derived from apical papilla [26]. The current study reports the cytotoxicity of SHEDs cultured in 0.5 mg/ml NaOCl after 24 hours of cultivation. This toxicity of NaOCl may be a result of its high pH, which disrupts the cytoplasmic membrane's stability [27]. Mantellini and colleagues discovered that stem cells directly exposed to 0.5 mg/ml NaOCl exhibited membrane disruption, which is indicative of cell necrosis [28]. Additionally, these data show that an agent's toxicity would grow gradually over time and at larger doses. This finding is consistent with earlier research, indicating that toxicity is time- and concentration-dependent [29].

ALP is a naturally occurring enzyme found throughout the body. When bones are developing or when bone cells are active, the level of this enzyme increases. ALP is involved in the regulation of cell growth and acts as an early indicator of osteogenic cell differentiation [18]. The current study assessed the osteogenic differentiation of SHEDs cultivated in varied concentrations of NaOCl using the ALP test. After seven days of incubation, the ALP activity of all test specimens, including the control, climbed after 14 days of incubation and then declined after 21 days of incubation. After seven days of incubation, the control had the lowest ALP activity. After 14 days of incubation, the maximum level of ALP activity was seen with the control, followed by 0.005 mg/ml NaOCl. When compared to the control, ALP levels in SHEDs grown in 0.0125 mg/ml and 0.005 mg/ml NaOCl increased after 21 days of incubation. DSPP is a parent protein that is synthesized by the craniofacial skeleton's connective tissues, notably bone and dentin, with a high gene expression level in the dentinal matrix. OCN, which is a protein, contains gamma-carboxyglutamic acid and is usually expressed throughout the end stages of osteoblast and odontoblast formation [30]. STRO-1 is a major odontoblast-originating marker for root dentin formation [31]. As a result, these genes were involved in the present trial. The expression levels of OCN and DSPP for SHEDs grown in varied concentrations of NaOCl rose after seven days of culturing, indicating that they are expressed in later stages of odontoblastic differentiation [30]. In the current study, the expression levels of the DSPP and OCN genes rose as the dilution of NaOCl dropped. In the current study, the expression levels of the STRO-1 gene dropped as the incubation duration rose and the dilution of NaOCl decreased. This observation might be explained by the fact that STRO-1 expression has previously been shown to fade with time in culture and as the number of cell passages rises [32].

The relative gene expression levels employed in this research are consistent with a study done by Liu and colleagues on stem cells derived from human permanent teeth, periodontal ligaments, and gingiva [9]. However, research by Martin and colleagues discovered that when lower amounts of NaOCl were utilized, the substantial negative effect on DSPP expression was reduced [8]. Irrigants, particularly NaOCl in large concentrations, are believed to denature dentin-derived growth factors, which would explain these findings [33]. DPSCs placed in scaffolds alone proliferated at slower rates and demonstrated lower levels of odontoblast-like markers in a tooth slice model during an in vivo study [34]. These results suggest that morphogens, such as the many tissue factors located in dentin, are sufficient to stimulate dental stem cell survival, proliferation, and, most importantly, differentiation. However, the above finding needs further investigation with respect to SHEDs.

The study's limitation was that it only tested the effect of SHEDs cultured in NaOCl for up to 24 hours for MTT and 21 days for ALP. Additionally, a long-term investigation is necessary, as the PCR results for the adhesion and differentiation factors are limited due to their measurement at certain periods. Additionally, an inquiry on a counteragent capable of neutralizing the toxicity of NaOCl is required, as is an investigation on scaffolding and growth regulators for better tissue engineering.

## Conclusions

Different doses of NaOCl other than 0.5 mg/ml revealed encouraging outcomes in terms of proliferation, long-term ALP functioning, and odontogenic differentiation potential when cultivated in SHEDs, given that regeneration methods need the survival of host stem cells. When cultured in SHEDs, 0.5mg/ml NaOCl exhibited cytotoxicity, decreased ALP activity, and a diminished capacity to differentiate compared to other dilutions of NaOCl. Based on these findings, lower dilution of NaOCl should be preferred in regenerative therapies involving SHEDs. To arrive at an agreement on the appropriate concentration of NaOCl for endodontic irrigation, further additional clinical research is required.
